# Motor skills and working memory capacity in preadolescents born very preterm

**DOI:** 10.1111/dmcn.70043

**Published:** 2025-10-23

**Authors:** Sebastian Ludyga, Markus Gerber, Martina Studer, Mark Brotzmann

**Affiliations:** ^1^ Department of Sport, Exercise and Health University of Basel Basel Switzerland; ^2^ Division of Neuropediatrics and Developmental Medicine University of Basel, University Children's Hospital Basel Switzerland

## Abstract

**Aim:**

To investigate the association of very preterm birth with visuospatial working memory in preadolescents and its mediation via motor skills and a neural index of working memory capacity.

**Method:**

Case–control matching based on sex and age resulted in 53 participants born before 32 weeks of gestation and 53 participants (24 males, 29 females) born at term. All participants performed a change detection task that assessed working memory capacity from their k‐score. The contralateral delay activity (CDA) elicited by the task was recorded using electroencephalography. Additionally, participants completed the Movement Assessment Battery for Children, Second Edition (MABC‐2).

**Results:**

Participants born very preterm showed lower k‐scores on the change detection task, lower negativity of the CDA as well as lower manual dexterity and balance on the MABC‐2. Based on structural equation modelling with bias‐corrected bootstrapping, poor motor skills fully mediated the association of very preterm birth with lower k‐scores. Similarly, very preterm birth had an indirect effect on CDA via motor skills.

**Interpretation:**

Children born very preterm face difficulties in maintaining visuospatial information because of a lower working memory capacity compared to peers born at term. Given the mediating role of motor skills, poor balance and manual dexterity in particular might serve as predictors of increased risk for prolonged impairments.

## Graphical abstract

Structural equation model showing the mediating effect of motor skills on the association of very preterm birth and working memory difficulties.

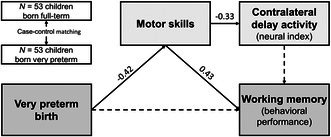

**This original article is commented by Hsieh on pages** 741‐742 **of this issue.**

**Abbreviations**
CBAT: children born at term
CBVP: children born very preterm
CDA: contralateral delay activity
MABC‐2: Movement Assessment Battery for Children, Second Edition
MVPA: moderate‐to‐vigorous physical activity

**What this paper adds**

- Very preterm birth is associated with lower working memory capacity in preadolescence.
- These deficits are accompanied by problems in adapting to working memory demands.
- Motor skills mediate preterm birth‐related impairments in visuospatial working memory.
- Poor balance and manual dexterity are risk factors for prolonged impairments.

The global prevalence of preterm birth is estimated at 9.9%, with 15% of the livebirths requiring intensive neonatal care, as they occur at less than 32 weeks of gestation.^1^ This specific gestational age is associated with an increased risk for injuries and altered development of the brain,^2^ with structural alterations (e.g. abnormalities in cortical thickness and surface area) affecting working memory.^3^ Children born very preterm (CBVP) face pronounced difficulties on tasks demanding visuospatial abilities in particular,^4^ whereas early difficulties in verbal working memory are compensated for by a catch‐up effect.^5^ Impairments in this cognitive domain persist until adulthood^6^ and partly underlie the relation between very preterm birth and poor academic performance,^7^ as well as lower occupational and socioeconomic achievement.^4^ Evidence on interventions that target preterm birth‐related working memory deficits directly by cognitive training is characterized by inconsistent results as well as a high risk of bias, and transfer‐effects of this type of training to other domains of health and performance have been questioned.^8^, ^9^ Advancing the understanding of factors underlying working memory deficits is crucial for the refinement of interventions and the development of alternative approaches.

In addition to poor visuospatial working memory, CBVP are characterized by moderate impairments in motor skills, independent of a history of cerebral palsy.^10^, ^11^ Deficits in both domains are interrelated, as cognitive function appears to be grounded in the ability to plan and control motor actions.^12^ In neurotypical children, their interrelation is indicated by an association of better working memory with higher locomotor skills, object control, and stability.^13^, ^14^ Neuroimaging findings support the idea of shared neural resources, since regions recruited for motor planning and preparation also contribute to working memory.^15^ Moreover, a conceptual model suggests that training motor skills with coordinative exercise elicits specific benefits for visuospatial working memory by acting on underlying cognitive control processes that undergo developmental changes.^16^

The contralateral delay activity (CDA) recorded with electroencephalography (EEG) serves as a neural index of working memory capacity and related attentional filtering.^17^ Its amplitude scales with the number of items maintained, with more pronounced differences as children develop.^18^ This component of event‐related potentials can be elicited by the encoding period in a change detection task that probes a memory array bilaterally. The calculation of the CDA as the difference between the attended and non‐attended hemifield allows the examination of mechanisms underlying working memory deficits independent of attentional problems that children born very preterm frequently face.^19^ In addition, experimental evidence underlines that the CDA increases after training that promotes the acquisition of complex motor skills.^20^ Consequently, this neural index has the potential to provide novel insights into the interrelation of deficits in motor skills and visuospatial working memory among CBVP.

Our study aimed to investigate the direct association of very preterm birth with visuospatial working memory and its potential mediation via motor skills in preadolescents. We further examined CDA as a secondary mediator of the relation between motor skills and working memory.

## METHOD

### Participants

CBVP were directly recruited from the University Children's Hospital Basel and the Kantonsspital Aarau between 2018 and 2022. During the same period, children born at term (CBAT) from the same regions were enrolled in a parallel study (led by the same project team) that used identical measures to be able to build matched pairs. Common inclusion criteria were: 9 to 13 years of age, right‐hand dominance (to reduce variation in neural activation patterns), corrected‐to or normal vision, as well as very preterm birth for cases (≤ 32 weeks of gestation), and term‐birth for controls (≥ 37 weeks of gestation). Participants with acute or chronic diseases that were contraindications for exercise and/or impaired the practicability of the scheduled exercise session were excluded. Further exclusion criteria were structural epilepsy and an IQ below 85 for CBVP and the presence of a mental disorder in CBAT. Written informed consent was obtained from legal guardians and all participants expressed their willingness to engage in the study. The study protocol was approved by the local ethics committee (Ethikkommission Nordwest‐ und Zentralschweiz) and followed the guidelines of the Declaration of Helsinki.

### Study design

After screening of the medical records, participants completed a laboratory visit lasting 90 to 120 minutes. They filled in the Family Affluence Scale, the Strengths and Difficulties Questionnaire, and the 7‐day physical activity recall protocol. Reported physical activity in different intensity levels was combined to yield the time spent in moderate‐to‐vigorous physical activity (MVPA). This variable was assessed as a potential confounder, since MVPA can contribute to motor skills. After a resting period, participants performed a computerized change detection task with simultaneous recording of brain activity via EEG. Anthropometric data (weight, height) was collected and participants completed the Movement Assessment Battery for Children, Second Edition (MABC‐2).

### Motor skills

The MABC‐2 is composed of age‐appropriate fine and gross motor tasks designed to evaluate motor proficiency in children aged 3 to 16 years.^21^ The battery consists of three items that evaluate hand–eye coordination and fine motor control (manual dexterity subscale), three items that assess the ability to maintain stability and control over their body during dynamic tasks (balance subscale), and two items measuring the ability to throw and catch objects (aiming and catching subscale). For each subscale, raw scores were calculated based on the individual performance, which were then converted into age‐ and sex‐adjusted standard scores.

### Cognitive task

The change detection task was administered with E‐Prime 3.0 (Psychology Software Tools, Pittsburgh, PA, USA; https://pstnet.com/). Each trial commenced with a fixation cross (600–700ms) and a cue (200 ms) providing information about whether the left or right hemifield had to be remembered (Figure S1). After a fixation (300–400 ms), a memory array (100 ms), which contained one or three coloured squares on each hemifield, was presented. For the retention period (900 ms), the stimuli were removed from the screen. The subsequent probe showed the squares from the cued hemifield in the same or a different colour. By pressing a button, participants had to indicate whether they detected a change. The coloured squares were presented against a black background, with distances to the fixation cross fixed on the x‐axis, but random variation (in a specified range) on the y‐axis. Two set lengths of the memory array were presented with an equal probability and in randomized order. In total, 224 trials were equally distributed across four blocks, which were preceded by a practice round with 16 trials. For each set size, average reaction times (on response‐correct trials) and accuracy rates were calculated. Moreover, k‐score was calculated as follows: set size*((hit rate–false alarm rate)/(1–false alarm rate)). The highest individual k‐score was used as main performance outcome.

### EEG recording and processing

Sixty‐four active electrodes (with positions corresponding to the 10:10 system) were mounted to the participant's head (using a flexible cap) to record the electroencephalogram during the change detection task. The ground electrode was positioned at AFz and Cz served as reference. All electrodes were filled with highly conductive gel to reduce impedance to 10 kΩ or lower. Data were amplified (ActiCHamp, BrainProducts, Gliching, Germany), band‐pass filtered and recorded at a sampling rate of 500 Hz.

Offline processing was performed with BESA Research 7.1 (Brain Electric Source Analysis, Gräfelfing, Germany). A detailed description of all steps is provided in Appendix S1. The procedure included artefact correction, artefact rejection based on amplitude thresholds, high‐pass as well as low‐pass filtering, and baseline‐correction. Segments (0–900 ms) were created and averaged within each condition. Contralateral activity was subtracted from the ipsilateral activity to derive the CDA for trials cueing either the left or right hemifield. The waveforms from both cue conditions were averaged with equal weighting. Separately for set size 1 and 3, the CDA amplitude was extracted as the average pooled over the parieto‐occipital region within the latency range from 250 ms to 600 ms.

### Statistical analyses

SPSS 29.0 and the AMOS 29.0 (IBM, Armonk, NY, USA) were employed for statistical analyses. Case–control matching (restricted to participants with complete data) was performed with the FUZZY plugin based on sex (exact match) and age (low tolerance). Based on sample size calculation, the minimum sample size was 78 (Appendix S1). The participants' characteristics (gestational age, anthropometrics, MVPA, psychopathological symptoms, socioeconomic status), MABC‐2 scores, cognitive performance, and CDA (within one and three item conditions) were compared between groups using a series of Student's *t*‐test. The association between birth status and working memory maintenance was examined with structural equation modelling employing the maximum likelihood method. The k‐score was predicted by group (coded 0 = CBAT; 1 = CBVP), while considering a potential indirect effect via MABC‐2. In addition, CDA_3_ was included as a second mediator for the association between MABC‐2 and k‐score. Only the CDA elicited from the high working memory load condition was included in the model, given that CBVP tend to show more pronounced impairments at increasing task difficulty.^4^ We used manifest variables for all factors, except MABC‐2, which was included as latent construct with its subscales serving as indicators. The models were controlled for variables, if potential confounders correlated with k‐scores based on zero‐order correlations. The bias‐corrected Bootstrap method (5000 samples) was applied to estimate the 95% confidence intervals for both standardized direct and indirect effects. Model fit was tested for all models and considered good (at root mean square error of approximation ≤0.08 and χ^2^/df ≤2).

## RESULTS

### Group differences

**FIGURE 1:**
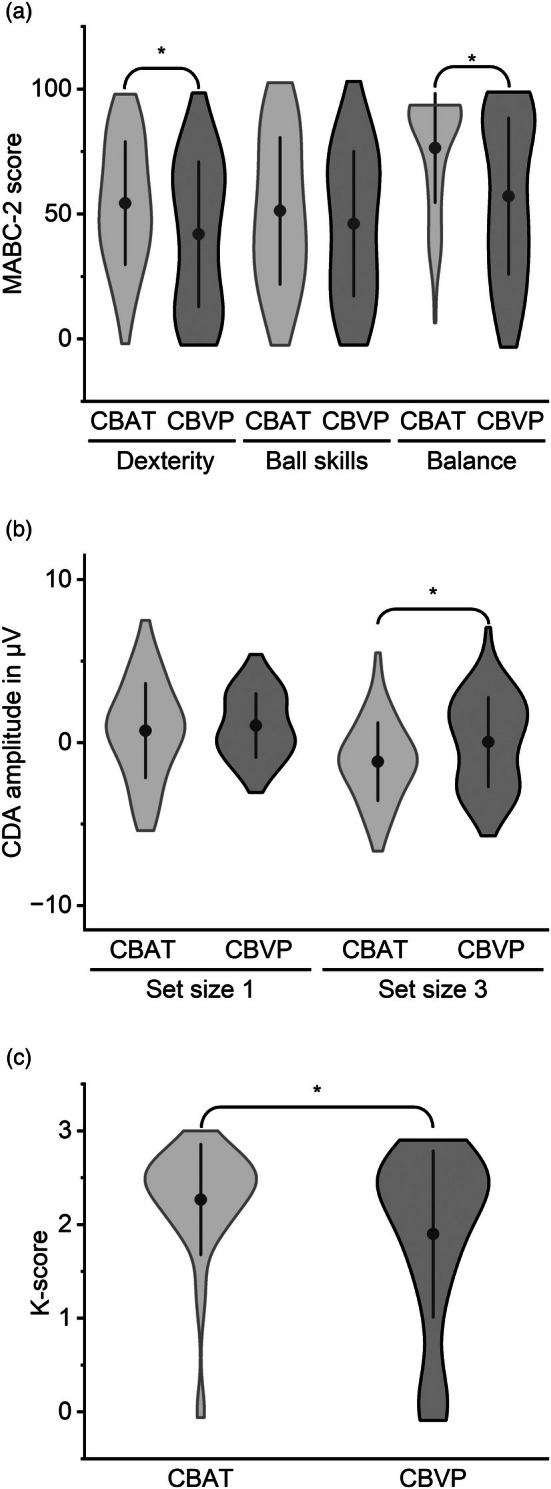
Violin plots comparing MABC‐2 score (a), CDA (b), and k‐score (c) between CBVP and CBAT. Notes: The width of each violin represents the kernel‐smoothed probability density of the data, the dot shows the mean, and the line represents the standard deviation. *p < 0.05 for group comparison using *t*‐test. Abbreviations: CBAT, children born at term; CBVP, children born very preterm; CDA, contralateral delay activity; MABC‐2, Movement Assessment Battery for Children, Second Edition.

FUZZY matching resulted in 106 participants, with an equal distribution of 24 males and 29 females per group. Compared to CBAT, CBVP had lower gestational age (*t*(104) = 26.81, *d* = 5.30, *p* < 0.001) and showed a tendency towards higher Strengths and Difficulties Questionnaire scores (*t*(104) = −1.94, *d* = −0.38, *p* = 0.055). In contrast, there were no group differences in anthropometrics (age, height, body mass index), socioeconomic status, MVPA levels, and reaction time on the change detection task (Table 1). CBVP showed lower scores on manual dexterity (*t*(104) = −2.38, *d* = −0.46, *p* = 0.019) and balance (*t*(104) = −3.70, *d* = −0.72, *p* < 0.001) relative to CBAT (Figure 1a). They further had lower negativity of the CDA_3_ (Figure 1b; *t*(104) = −2.40, *d* = −0.47, *p* = 0.018), and lower k‐score (Figure 1c; *t*(104) = 2.51, *d* = 0.49, *p* = 0.014).

**TABLE 1:** Comparison of anthropometrics, physical activity, socioeconomic status, psychopathological symptoms, and reaction time on the change detection task between groups.

	CBAT (*n* = 53)		CBVP (*n* = 53)^a^		*t*‐test		
	Mean	SD	Mean	SD	*T*	*d*	*p*
Weeks of gestation	39.1	1.5	29.6	2.1	26.8	5.30	<0.001
Age in years:months	11.0	1.7	11.0	1.10	−0.03	−0.01	0.975
Height in cm	148.4	11.5	145.1	12.3	1.42	0.28	0.158
BMI in kg/m^2^	17.5	2.5	17.1	2.6	0.73	0.14	0.468
MVPA in min/d	49.0	32.4	51.7	58.3	−0.30	−0.06	0.765
FAS score	6.5	1.8	6.8	1.5	−0.85	−0.17	0.396
SDQ score	10.1	4.1	11.8	5.0	−1.94	−0.38	0.055
RT (1 item) in ms	970.6	273.5	1015.5	301.8	−0.80	−0.16	0.426
RT (3 items) in ms	1133.1	342.3	1134.7	322.7	−0.02	0.00	0.981

Abbreviations: BMI, body mass index; CBAT, children born at term; CBVP, very preterm born children; FAS, Family Affluence Scale; MVPA, moderate‐to‐vigorous physical activity; SD, standard deviation; SDQ, Strengths and Difficulties Questionnaire; RT, reaction time on the change detection task.

^a^ Eight born extremely preterm and 45 born very preterm.

### Mediation model

**FIGURE 2:**
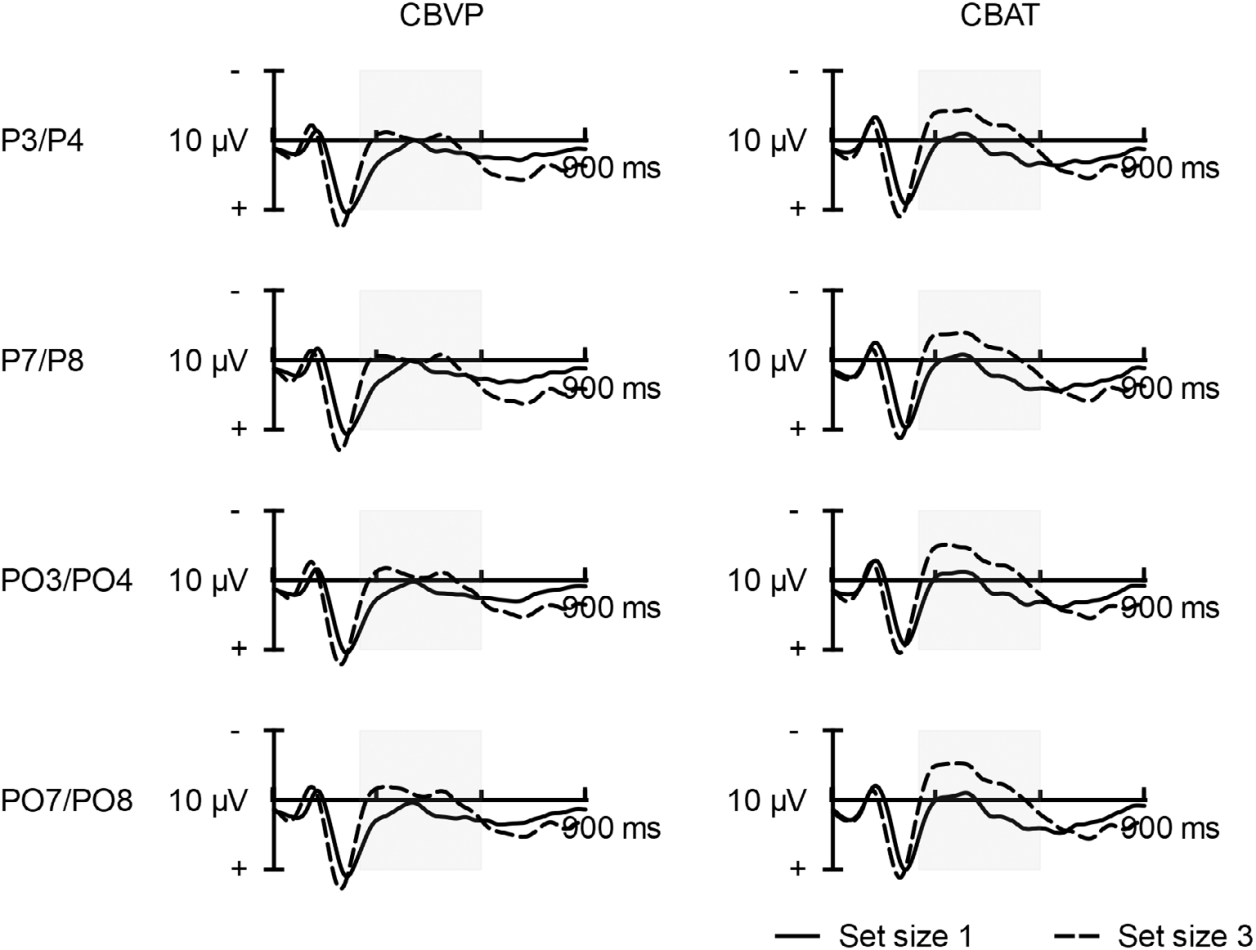
Event‐related potential waveforms (ipsilateral subtracted from contralateral activity) displayed for set size 1 and 3 in CBVP and CBAT groups. Note: The vertical grey bar indicates the latency range used to calculate the contralateral delay activity. Abbreviations: CBAT, children born at term; CBVP, children born very preterm.

**FIGURE 3:**
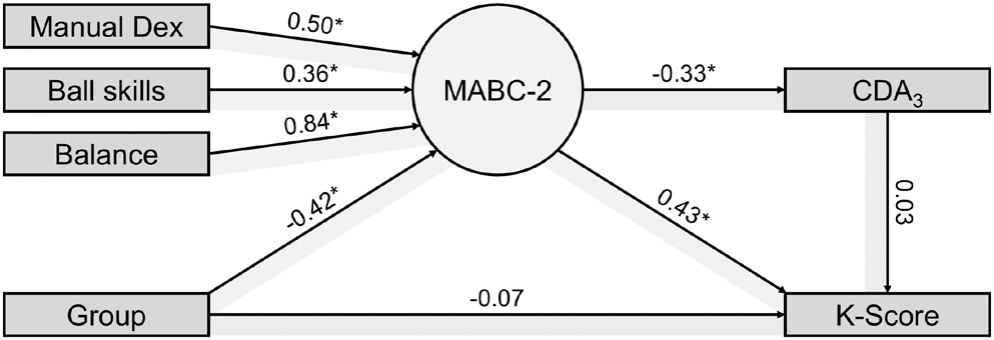
Structural equation model investigating the association of group and k‐scores, as well as mediating effects of motor skills and CDA. Note: Group is coded 0 = children born at term, 1 = children born very preterm. Abbreviations: CDA_3_, contralateral delay activity elicited by a memory array containing three items; Dex, dexterity; MABC‐2, Movement Assessment Battery for Children, Second Edition.

The event‐related potential waveforms for both set sizes are displayed separately for CBVP and CBAT in Figure 2. Preliminary analysis did not support a statistically significant correlation of MVPA levels, socioeconomic status, and body mass index with k‐scores (*r*(357) ≤ 0.14, *p* ≤ 0.151), so that they were not accounted for. In the SEM model, CBVP was associated with a lower MABC‐2 score (Figure 3; β = −0.42, *p* = 0.003). Lower scores on MABC‐2 were further associated with a lower k‐score (β = 0.43, *p* = 0.005) and negativity of the CDA_3_ (β = −0.33, *p* = 0.009). In contrast, there was no relation of group and k‐score as well as CDA_3_ and k‐score. The fit of the model to the data was good (root mean square error of approximation = 0.008, χ^2^/df = 1.01).

When the model was estimated with 5000 bootstrap resamples (Table 2), direct effects of group on MABC‐2 score as well as MABC‐2 score on both k‐score and CDA were revealed. The group association with k‐scores and CDA was better explained by an indirect effect via MABC‐2. In contrast, CDA did not mediate the association of MABC‐2 and k‐scores.

**TABLE 2:** Direct effects of group on k‐score and indirect effects via motor skills and CDA.

	Variables	Lower 95% CI	Upper 95% CI	*p*
Standardized direct effect	Group → MABC‐2	−0.64	−0.18	0.001
	MABC‐2 → CDA_3_	−0.53	−0.12	0.004
	MABC‐2 → k‐score	0.11	0.81	0.008
	CDA_3_ → k‐score	−0.19	0.26	0.818
Standardized indirect effect	Group → MABC‐2 → CDA_3_	0.04	0.32	0.003
	Group → MABC‐2 → k‐score	−0.50	−0.04	0.004
	MABC‐2 → CDA_3_ → k‐score	−0.11	0.06	0.745

*Note*: The bias‐corrected centile method with estimation of 5000 bootstrapping samples was used.

Abbreviations: CDA_3_, contralateral delay activity elicited by a memory array containing three items; CI, confidence interval; MABC‐2, Movement Assessment Battery for Children, Second Edition.

## DISCUSSION

In comparison to CBAT, CBVP showed less pronounced negativity of the CDA for three items. This difference was accompanied by a lower capacity to store visuospatial information, which was mediated by lower motor skills. Moreover, the association between motor skills and working memory capacity was direct and not better explained by CDA.

Our findings are in line with previous observations underlining that very preterm birth continues to affect visuospatial working memory at the transition from childhood to adolescence.^5^ Lower performance on the change detection task in CBVP was evident by a k‐score that reflected the ability to maintain less than two items in working memory, whereas CBAT showed a storage capacity exceeding two items. Given that reaction times on probes after one‐ and three‐item memory arrays did not differ between groups, there was no indication for a speed‐accuracy trade‐off. This also implies that working memory is not confounded by impaired processing speed, even though evidence has partly attributed abnormalities in higher‐order cognitive functions among CBVP to delayed responses.^22^

The behavioural performance differences between groups were accompanied by a lower CDA in the three‐ item condition. Considering the lack of differences at one item level, this provides an indication that abnormalities related to very preterm birth can be identified at increased working memory load. In contrast, CBAT showed a set‐size related rise in the CDA amplitude, which reflects a higher capacity to store visuospatial information rather than the sensitivity to increased difficulty. This is supported by the development of an asymptote detectable at the point, where no additional items can be encoded.^23^ As an index of the capacity and access to working memory, the CDA has a predictive value for interindividual differences in a wide range of cognitive performance domains.^17^ However, a lower negativity of the CDA in CBVP was not associated with lower k‐score, suggesting that processes indexed by this component did not underlie group differences in performance on the change detection task. Conversely, these behavioural and event‐related potential indices represent two distinct abnormalities associated with very preterm birth. The lower CDA points to problems regarding the retention of items, which complements findings underlining immature patterns of recruitment of brain networks during the maintenance of visuospatial information.^24^ Moreover, the CDA results from the processing of object‐identity information (i.e. coloured squares),^25^ so that our findings suggest difficulties in remembering items of the memory array rather than their location in CBVP. In contrast, behavioural performance on visual array tasks do not allow a differentiation between object‐identity and location information and is affected by attentional control, which does not necessarily reflect specific working memory abilities. Therefore, a lower k‐score may partly by attributed to common difficulties in executive function among CBVP, which encompass the top‐down control of attention.^26^

Regarding motor skills, CBVP scored lower on the MABC‐2 compared to CBAT, with pronounced differences in manual dexterity and balance. Impaired motor skills fully mediated the association of very preterm birth with low visuospatial working memory. This finding can be interpreted from the perspective of an embodied cognition, which assumes that the body and its functions shape the nature of cognitive activity and the content of the processed representations.^27^ This view further stresses that interaction with the environment creates possibilities for action and, in turn, that new motor skills advance opportunities for exploration and learning. Consequently, early motor disabilities due to very preterm birth may limit the interacting with the environment, which is likely to affect the development and differentiation of cognitive abilities. This is supported by longitudinal findings linking poor motor skills in infants with low performance in different cognitive domains.^28^ These links have been assumed to be specific,^27^ so that the acquisition and refinement of selected motor skills will propel the development of specific rather than domain‐general cognitive abilities. In this respect, our results underscore that CBVP with higher motor skills show lower difficulties with storing visuospatial information in particular.

The association between motor skills and working memory was not mediated by cognitive control processes indexed by the CDA, even though higher motor skills were moderately related to a higher negativity of this event‐related potential component. As CDA did not explain variance in behavioural performance on the change detection task, the association of motor skills with both behavioural and neuroelectric outcomes may not necessarily be because of a common underlying mechanism. Based on source localization, the superior parietal lobule (Brodmann area 7) has been identified as the main generator of the CDA.^17^ Functional differentiation of portions of the superior parietal lobule revealed that the parieto‐occipital sulcus is employed for the analysis of visual motion cues useful for object motion detection (during self‐motion and spatial navigation), whereas more anterior parts are recruited during visuomotor control of limb actions.^29^ Meta‐analytical findings suggest that the activity of the superior parietal lobule decreases with the progression of motor learning, suggesting an increased efficiency over time.^30^ Consequently, the interrelation of motor skills and the CDA may be attributed to a joint neural substrate. In turn, this also implies that the alterations in cortical thickness related to preterm birth^3^ and decreased local network efficiency in the superior parietal lobe with shorter gestation^31^ might explain differences in both motor skills and the maintenance of visuospatial information in working memory between CBAT and CBVP.

While our findings underscore motor skills as a mediator of very preterm birth‐related impairments in working memory capacity, the cross‐sectional design of the study limits predictions on the effectiveness of interventions targeting motor skills. Similarly, the direction of the effects proposed in our mediation model cannot be verified by data collected from a single time point. Moreover, our study provided novel insights into working memory processes affected by very preterm birth, but these processes did not underlie the association between motor skills and k‐score. This indicates that poor behavioural performance in CBVP was due to mechanisms that we did not examine in our study. For example, the interrelation between motor skills and k‐scores could also be driven by abnormalities in the encoding and retrieval of visuospatial information that are not indexed by the CDA, or by a diminished learning effect across both tasks. These abnormalities might also be attributed to a higher prevalence of neurodevelopmental disorders in CBVP, as we did not control for their occurrence within our sample. Additionally, we investigated very preterm birth‐related deficits in visuospatial working memory capacity by comparison with CBAT. The different recruitment strategies required for the groups might have increased the risk of a selection bias. Because of the specific group comparison, it remains unclear whether there is a linear relation between working memory deficits and gestational age and whether motor skills have a mediating role across all preterm groups.

### CONCLUSION

At the transition to adolescence, CBVP face difficulties in maintaining visuospatial information. On a neurocognitive level, impaired capacity to store such information is indexed by the inability to adapt cognitive control to higher working memory load. The deficits in this cognitive domain are mediated by motor skills. Poor balance and manual dexterity in particular might serve as predictors of increased risk for prolonged impairments in visuospatial working memory and call for early intervention.

## CONFLICT OF INTEREST

None.

## Supporting information


**Appendix S1:** Sample size calculation and EEG processing.

**Figure S1:** Trial procedure of the change detection paradigm.

## Data Availability

Data will be made available on request to the corresponding author.
