# Supplementary material for: Motor skills and working memory capacity in preadolescents born very preterm
Source: Dev Med Child Neurol. 2025 Oct 23;68(6):784–91. doi: 10.1111/dmcn.70043 (PMC13160396; doi:10.1111/dmcn.70043)
Supplement: Supplementary file 1 — Appendix S1: Sample size calculation and EEG processing. [file DMCN-68-784-s001.docx]

**Appendix S1**

**Sample size calculation**

Due to a lack of studies investigating the association of preterm birth with working memory and its mediation by motor skills, sample size was calcaluted for individual regression paths. A previous comparison of FTC and VPBC resulted in small-to-moderate difference in visuospatial working memory (*d* = 0.27 to 0.51 depending on the selected outcome, which equals a converted *R* ≥ 0.13).^1^ Meta-analytical findings in typically-developing children have reported small-to moderate associations between motor skills and working memory, with values spanning from *R* = 0.18 to *R* = 0.20.^2,3^ As values for VPBC were not available from the exisiting literature, we expected a similar or even higher association between motor skills and working memory in this group. Based on *R* = 0.13 (lowest strength of association) and *p* = 0.05, 78 participants were required to achive 85 % power.

**EEG processing**

For offline processing, data was imported in BESA Research 7.1 (Brain Electric Source Analysis, Germany). Virtual HEOG and VEOG channels informed automatic adaptive artefact correction was applied. The resulting data was high-pass filtered (forward phase shift of 0.1 Hz; slope 6 dB/octave) and baseline-corrected (-200 ms to stimulus onset). Artefacts that remained after the correction procedure were rejected using individual gradient and amplitude thresholds to account for interindividual differences. Subsequently, segments spanning the period from onset of the memory array to 900 ms were averaged separately for the two set sizes. The resulting segments (1 item: 85.1 ± 15.2; 3 items: 71.2 ± 16.2) were low-pass filtered (zero-phase shift of 30 Hz; slope 24 dB/octave) and re-referenced to the average of all channels. The average reference was used to avoid that reference and region of interest are close to each other^4^ and to allow comparability with previous studies investigating the CDA.^5^ Contralateral activity was subtracted from the ipsilateral activity to derive the CDA for trials cueing either the left or right hemifield.^6^ The waveforms resulting for both cue conditions were averaged based on an equal weighting. Separately for set size 1 and 3, the CDA amplitude was extracted as the average pooled over the parieto-occiptal region (left/right hemisphere: P3/4, P7/P8, PO3/PO4, PO7/PO8) within the latency range from 250 to 600 ms following the onset of the memory array.

**References**

1. Woodward LJ, Horwood LJ, Darlow BA, Bora S. Visuospatial working memory of children and adults born very preterm and/or very low birth weight. *Pediatr Res*. 2022;91(6):1436-1444. doi:10.1038/s41390-021-01869-w

2. Bao R, Wade L, Leahy AA, et al. Associations Between Motor Competence and Executive Functions in Children and Adolescents: A Systematic Review and Meta-analysis. *Sports Med*. 2024;54(8):2141-2156. doi:10.1007/s40279-024-02040-1

3. Gandotra A, Csaba S, Sattar Y, et al. A Meta-analysis of the Relationship between Motor Skills and Executive Functions in Typically-developing Children. *J Cogn Dev*. 2022;23(1):83-110. doi:10.1080/15248372.2021.1979554

4. Yao D, Qin Y, Hu S, Dong L, Bringas Vega ML, Valdés Sosa PA. Which Reference Should We Use for EEG and ERP practice? *Brain Topogr*. 2019;32(4):530-549. doi:10.1007/s10548-019-00707-x

5. Feldmann‐Wüstefeld T. Neural measures of working memory in a bilateral change detection task. *Psychophysiology*. 2021;58(1):e13683. doi:10.1111/psyp.13683

6. Luria R, Balaban H, Awh E, Vogel EK. The contralateral delay activity as a neural measure of visual working memory. *Neurosci Biobehav Rev*. 2016;62:100-108. doi:10.1016/j.neubiorev.2016.01.003
