# Supplementary figures and images for: Motor skills and working memory capacity in preadolescents born very preterm
Source: Dev Med Child Neurol. 2025 Oct 23;68(6):784–91. doi: 10.1111/dmcn.70043 (PMC13160396; doi:10.1111/dmcn.70043)

**Supplement**


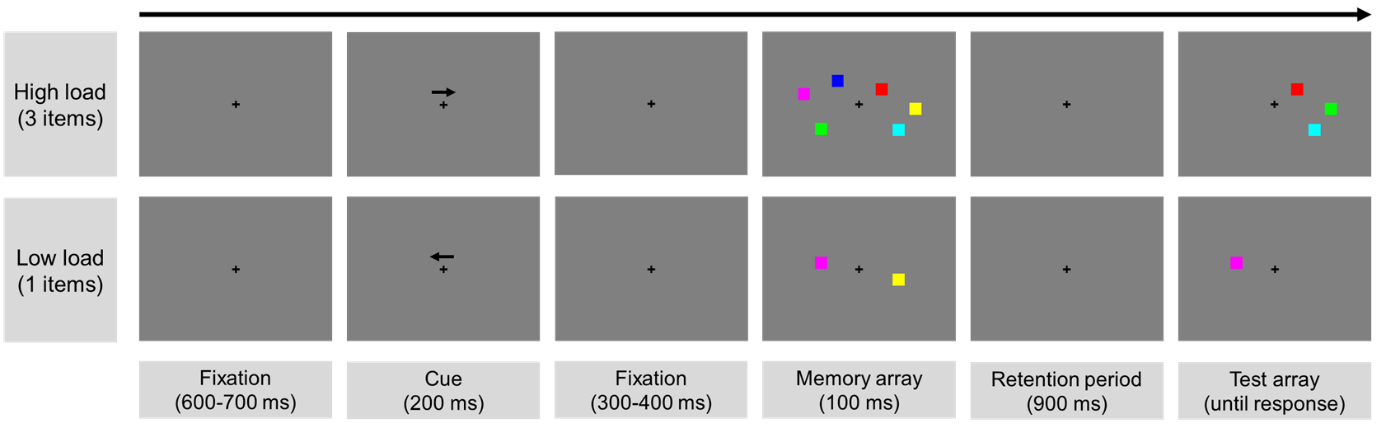


**Fig. S1.** Trial procedure of the Change Detection paradigm.

Supplement: Supplementary file 2 — Figure S1: Trial procedure of the change detection paradigm. [file DMCN-68-784-s002.docx]
